# Wundbehandlung nach dermatochirurgischen Eingriffen

**DOI:** 10.1007/s00105-026-05767-2

**Published:** 2026-09-01

**Authors:** Conrad Hempel, Till Mittank-Weidner, Thomas Volz, Lukas Kofler, Sonja Dengler, Sylke Schneider-Burrus, Viktor Schnabel, Franziska Jochims, Sonja Grunewald

**Affiliations:** 1 Klinik für Dermatologie, Venerologie und Allergologie, Universitätsklinikum Leipzig, Philipp-Rosenthal-Str. 23, 04103 Leipzig, Deutschland; 2 Klinik für Dermatologie und Allergologie, TUM Klinikum rechts der Isar, School of Medicine and Health, Technische Universität München, München, Deutschland; 3 MVZ Hautzentrum am Holzmarkt, Corius Biberach GmbH, Biberach an der Riß, Deutschland; 4 Klinik für Dermatologie, Klinikum Dortmund GmbH, Universität Witten/Herdecke, Herdecke, Deutschland; 5 Zentrum für Dermatochirurgie, Havelklinik Berlin, Berlin, Deutschland

**Keywords:** Postoperative Wundversorgung, Wundinfektion, Wundheilungsstörung, Evidenzbasierte Wundtherapie, Ästhetisch-funktionelles Ergebnis, Postoperative wound care, Wound infection, Impaired wound healing, Evidence-based wound therapy, Esthetic–functional outcomes

## Abstract

**Hintergrund:**

Die postoperative Wundversorgung ist ein wesentlicher Bestandteil dermatochirurgischer Eingriffe und beeinflusst den Heilungsverlauf sowie das ästhetisch-funktionelle Ergebnis. In der klinischen Praxis bestehen deutliche Unterschiede hinsichtlich eingesetzter Antiseptika, Verbandsmaterialien und Nachsorgekonzepte. Belastbare Daten zum tatsächlichen Vorgehen in der Dermatochirurgie in Deutschland sind bislang limitiert.

**Ziel der Arbeit:**

Ziel dieser Untersuchung war es, das aktuelle Vorgehen in der postoperativen Wundversorgung von operativ tätigen DermatologInnen in Deutschland zu erfassen und mit evidenzbasierten Empfehlungen abzugleichen.

**Material und Methoden:**

Es wurde eine anonyme, internetbasierte Umfrage mit 26 Fragen zur postoperativen Wundversorgung durchgeführt. Der Fragebogen wurde an alle Mitglieder der Deutschen Gesellschaft für Dermatochirurgie versendet und zusätzlich während der Jahrestagung 2025 angeboten. Die Auswertung erfolgte deskriptiv.

**Ergebnisse:**

Insgesamt wurden 62 vollständig ausgefüllte Fragebögen analysiert. Präoperativ sowie bei Verbandswechseln wurden überwiegend Octenidin-haltige oder alkoholbasierte Antiseptika eingesetzt. Die postoperative Versorgung nach primärem Wundverschluss war überwiegend homogen, während sich bei sekundär heilenden Wunden eine hohe Variabilität der verwendeten Verbandsmaterialien zeigte. Topische Antibiotika wurden trotz des Risikos der Resistenzentwicklung weiterhin in relevantem Umfang verwendet.

**Diskussion:**

Die Ergebnisse zeigen eine ausgeprägte Heterogenität der postoperativen Wundversorgung und eine nur teilweise Umsetzung evidenzbasierter Empfehlungen. Insbesondere der routinemäßige Einsatz topischer Antibiotika und Antiseptika bei nicht infizierten Wunden erscheint kritisch.

## Abstract (en)

**Background:**

Postoperative wound care is an essential component of dermatologic surgical procedures and has a significant impact on wound healing as well as esthetic and functional outcomes. In clinical practice, considerable differences exist with regard to the antiseptics used, dressing materials, and follow-up care concepts. Robust data on current postoperative wound care practices in dermatologic surgery in Germany are limited.

**Objectives:**

The aim of this study was to assess current postoperative wound care practices among skin surgeons in Germany and to compare them with evidence-based recommendations.

**Materials and methods:**

An anonymous, internet-based survey comprising 26 questions on postoperative wound care was conducted. The questionnaire was distributed to all members of the German Society for Dermatologic Surgery and was additionally offered during the 2025 annual meeting. Data were analyzed descriptively.

**Results:**

A total of 62 fully completed questionnaires were analyzed. Octenidine-containing or alcohol-based antiseptics were predominantly used both preoperatively and during dressing changes. Postoperative wound care following primary wound closure was largely homogeneous, whereas a high variability in dressing materials was observed for wounds healing by secondary intention. Topical antibiotics continued to be used to a significant extent despite the risk of developing resistance.

**Conclusion:**

The results demonstrate substantial heterogeneity in postoperative wound care and only partial implementation of evidence-based recommendations. In particular, the routine use of topical antibiotics and antiseptics in noninfected wounds appears questionable.

## Hintergrund und Fragestellung

Die postoperative Wundversorgung stellt einen zentralen Bestandteil der dermatochirurgischen Behandlung dar und beeinflusst maßgeblich sowohl den Heilungsverlauf als auch das ästhetisch-funktionelle Ergebnis. Trotz zahlreicher verfügbarer Publikationen bestehen in der klinischen Praxis teils erhebliche Unterschiede hinsichtlich verwendeter Materialien, Verbandswechselintervalle und Nachsorgekonzepte.

Während einzelne Studien spezifische Aspekte der Wundbehandlung untersucht haben, fehlen bislang umfassende Daten zum tatsächlichen Vorgehen von operativ tätigen DermatologInnen in Deutschland. Unklar ist, inwieweit evidenzbasierte Strategien umgesetzt werden, welche Präferenzmuster in der täglichen Routine dominieren und welche Herausforderungen Ärztinnen und Ärzte in der postoperativen Versorgung sehen.

Ziel der vorliegenden Untersuchung ist es daher, das derzeitige Vorgehen in der postoperativen Wundversorgung innerhalb der Deutschen Gesellschaft für Dermatochirurgie systematisch zu erfassen. Durch die Analyse der aktuellen Praxisrealität sollen mögliche Versorgungsvariationen identifiziert, diese mit Studiendaten abgeglichen werden und Ansatzpunkte für zukünftige Optimierungsstrategien abgeleitet werden.

## Studiendesign und Untersuchungsmethoden

Es wurde eine internetbasierte Umfrage mit insgesamt 26 Fragen zur postoperativen Wundversorgung entwickelt (Tab. 1). Der Link zum Fragebogen wurde einmalig an alle Mitglieder der Deutschen Gesellschaft für Dermatochirurgie (DGDC e. V.) versendet und war vom 10.10–19.11.25 nutzbar. Weiterhin bestand die Möglichkeit, während der Jahrestagung der DGDC e. V. in München vom 10.–12.10.25 einen QR-Code zu scannen und ebenfalls an der Studie teilzunehmen. Da keinerlei personenbezogene Daten erhoben wurden, erfolgte die Teilnahme anonym. Alle Fragen waren optional und konnten unbeantwortet bleiben. Die Umfrage wurde durch die Dermatological Study Initiative (DESSI) gefördert.

**Tab. 1:** DGDC-Mitgliederbefragung zur Wundbehandlung in der Dermatochirurgie

Einrichtung	( ) Klinik, ( ) Niederlassung	
*A*	*Antiseptika****:***	
	1.	Welches Antiseptikum verwenden Sie präoperativ standardmäßig?
	2.	Falls Sie in besonderen Situationen (z. B. schleimhautnah) abweichende Antiseptika nutzen, geben Sie bitte Indikation und Produkt an:
	3.	Welches Antiseptikum verwenden sie regelhaft zur Spülung von Wundhöhlen?
	4.	Weichen sie hiervon ab, sollte Knorpel, Knochen oder Sehne freiliegen?
	5.	Welches Wundprodukt legen Sie in Abszesshöhlen (als Tamponade) ein?
*B*	*Alloplastische Defektdeckung:*	
	1.	Welche Wundversorgung verwenden Sie nach Tumorresektion zur alloplastischen Defektdeckung standardmäßig?
	2.	Falls Sie in besonderen Situationen hiervon abweichen (z. B. freiliegender Knorpel, Knochen, Muskel, Sehne), geben Sie bitte Indikation und Produkt an:
*C*	*Primärer Wundverschluss und Lappenplastik:*	
	1.	Welche Wundversorgung verwenden Sie unmittelbar nach einem primären Wundverschluss oder einer Lappenplastik?
	2.	Welches Antiseptikum verwenden Sie bei den Verbandswechseln?
	3.	Welche Wundauflage verwenden Sie bei den Verbandswechseln?
	4.	In welchen Abständen wechseln Sie den Wundverband bis zum Fadenzug?
*D*	*Sekundäre Wundheilung und Wundkonditionierung:*	
	1.	Welche Wundversorgung verwenden Sie unmittelbar postoperativ nach Tumorexzision bei Wunden, die sekundär heilen oder granulieren sollen?
	2.	Welches Antiseptikum verwenden Sie bei den weiteren Verbandswechseln?
	3.	Welche Wundauflage verwenden Sie bei den Verbandswechseln?
	4.	In welchen Abständen wechseln Sie den Wundverband?
	5.	Weichen Sie von Ihrem Vorgehen bei Exzision von entzündlichen Hautveränderungen (z. B. Pilonidalsinus, Hidradenitis suppurativa) ab?
	6.	Setzen Sie Hilfsmittel bei sekundär heilenden oder wundkonditionierten Wunden regelhaft ein (z. B. elektives Débridement im Verlauf, Kaltplasmabehandlung, Unterdruckwundtherapie)? Wenn ja, welche?
*E*	*Hauttransplantation***:**	
	1.	Welche Wundauflage verwenden Sie auf das frisch transplantierte Spalthauttransplantat bzw. Vollhauttransplantat?
	2.	Legen Sie in der Regel einen Überknüpfverband an? Wenn ja: Nach welcher Zeit entfernen Sie den Überknüpfverband?
	3.	Welche Wundauflage verwenden Sie für die Verbandswechsel?
	4.	In welcher Frequenz führen Sie die Verbandswechsel durch?
	5.	Welches Antiseptikum verwenden Sie bei den Verbandswechseln?
	6.	Welche Wundauflage verwenden Sie unmittelbar postoperativ am Entnahmeareal?
	7.	Benutzten Sie regelmäßig Dermisersatzpräparate? Wenn ja, welche und bei welcher Indikation?
*G*	*Postoperative Wundinfektionen:*	
	1.	Welche Wundversorgung wählen Sie bei geschlossenen infizierten Wunden?
	2.	Welche Wundversorgung wählen Sie bei dehiszenten oder offenen infizierten Wunden?

Die technische Umsetzung der Online-Umfrage wurde von der Firma Transparent Werbeagentur GbR (Chemnitz) betreut. Grundlage des Fragebogens war ein Formular im Content-Management-System Contao. Die ausgefüllten Fragebögen wurden direkt an die Agentur und die Studienleitung (Erstautor) übermittelt.

Die explorative statistische Auswertung sowie die grafische Aufbereitung der Daten erfolgten mithilfe von Microsoft® Excel (Version 12.0; Redmond, WA, USA) und IBM SPSS Statistics Version 29 (IBM SPSS, Armonk, NY, USA). Die offenen Fragen des Fragebogens ließen aufgrund der heterogenen Antworten keine sinnvolle Kategorienbildung zu. Daher konnten sie nicht statistisch analysiert werden und wurden stattdessen einzeln aufgeführt und diskutiert.

## Ergebnisse

**Abb. 1:**
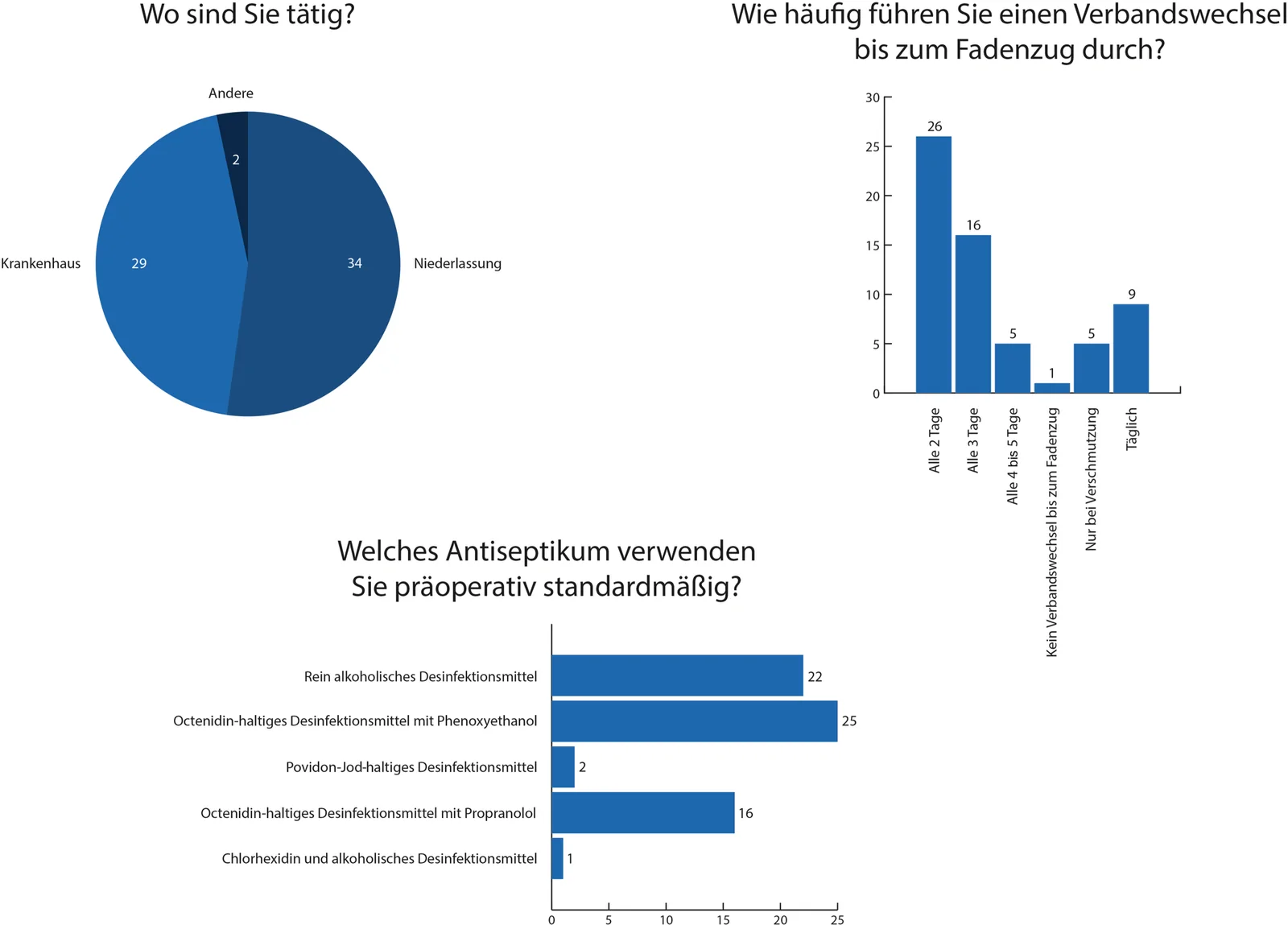
Übersicht über die Umfrageteilnehmenden und deren Standardverfahren in der perioperativen Wundversorgung. Gezeigt werden das Tätigkeitsfeld, die Häufigkeit von Verbandswechseln bis zum Fadenzug sowie die standardmäßig verwendeten präoperativen Hautantiseptika. Angegeben sind absolute Häufigkeiten

Zum Ende des Umfragezeitraums lagen insgesamt 62 vollständig ausgefüllte Fragebögen vor. Die Umfrage wurde per E‑Mail an 1367 Mitglieder versendet, was einer Rücklaufquote von 4,5 % entspricht. Die Hälfte der Teilnehmenden (*n* = 31; 50,0 %) gab an, in der Niederlassung tätig zu sein, während 29 Personen (46,8 %) für ihre jeweilige Klinik sprachen. Zwei Teilnehmende (3,2 %) wählten die Kategorie „sonstige Tätigkeit“ (Abb. 1).

### Verwendung von Antiseptika

Die präoperativ am häufigsten eingesetzten Antiseptika waren ausschließlich Octenidin-haltige Desinfektionsmittel (*n* = 22), gefolgt von ausschließlich alkoholischen Präparaten (*n* = 19) sowie kombinierten Octenidin- und alkoholhaltigen Präparaten (*n* = 14). In lediglich einem Fall wurde standardmäßig ein halogenhaltiges Desinfektionsmittel als Monopräparat verwendet.

Im schleimhautnahen Bereich wurden bevorzugt Octenidin-haltige Desinfektionsmittel eingesetzt. Einzelne Teilnehmende gaben an, Wasserstoffperoxid-haltige Desinfektionsmittel zu verwenden sowie bei freiliegendem Knorpel oder exponierten Sehnen auf Polyhexanid-haltige Präparate auszuweichen.

Am häufigsten werden Abszesshöhlen mittels Povidon-Jod-haltigen Desinfektionsmittels gespült (*n* = 29; 46,8 %), gefolgt von Polyhexanid-haltiger Wundspüllösung (*n* = 9; 14,5 %). Fünf Teilnehmende der Umfrage gaben an, zur Spülung Octenisept® (Schülke & Mayr GmbH, Norderstedt, Deutschland) zu verwenden (8,1 %). Sieben Teilnehmende (11,3 %) berichteten über den standardmäßigen Einsatz antibiotikahaltiger Gazen oder getränkter Streifen in Abszesshöhlen.

### Alloplastische Defektdeckung bei mehrzeitigen Eingriffen

Die mit Abstand am häufigsten genannte alloplastische Defektdeckung stellt Epigard (Biovision GmbH, Deutschland) dar (*n* = 11; 17,7 %), gefolgt von Suprathel (PolyMedics Innovations GmbH, Deutschland) (*n* = 4; 6,5 %) und Integra (Integra LifeSciences, USA) (*n* = 2; 3,2 %). In 3 Fällen (4,8 %) wurde über die Verwendung antibiotikahaltiger Salben in Kombination mit einer Fettgaze berichtet. Darüber hinaus lagen zahlreiche Einzelnennungen vor.

### Primärer Wundverschluss und Lappenplastik

Die überwiegende Mehrheit der Teilnehmenden verwendete zur unmittelbaren postoperativen Wundversorgung nach primärem Wundverschluss Pflaster bzw. Nahtpflaster, ggf. in Kombination mit einer Gaze, einer Kompresse oder einem aluminiumbedampften Vliesstoff. Drei Teilnehmende (4,8 %) gaben an, regelhaft antibiotikahaltige Salben einzusetzen. Zwei Teilnehmende (3,2 %) verwendeten standardmäßig Sprühpflaster, während ein Teilnehmender (1,6 %) angab, auf eine postoperative Wundversorgung zu verzichten.

Als Standarddesinfektionsmittel bei Verbandswechseln wurden überwiegend Octenidin-haltige Desinfektionsmittel verwendet (*n* = 36; 58,0 %), gefolgt von alkoholischen Desinfektionsmitteln (*n* = 18; 29,0 %). Sechs Teilnehmende (9,7 %) gaben an, bei Verbandswechseln regulär kein Desinfektionsmittel einzusetzen.

Der erste Verbandswechsel erfolgte am häufigsten im Abstand von 2 Tagen (*n* = 26; 41,9 %), gefolgt von einem Wechsel alle 3 Tage (*n* = 16; 25,8 %) sowie täglichen Wechseln (*n* = 9; 14,5 %). Fünf Teilnehmende (8,1 %) wechselten den Wundverband ausschließlich bei Verschmutzung, während ein Teilnehmender bis zum Fadenzug keinen Verbandswechsel durchführte.

### Sekundäre Wundheilung und Wundkonditionierung

Die unmittelbare postoperative Wundversorgung nach Tumorexzision bei sekundär heilenden bzw. granulierenden Wunden zeigte eine hohe Heterogenität und beruhte überwiegend auf Einzelnennungen. Ein Großteil der Teilnehmenden verwendete ein Verbandskonzept unter Einbeziehung von Gaze (*n* = 27; 43,5 %). Schaumverbände kamen ebenfalls häufig zum Einsatz (*n* = 11; 17,7 %), ebenso Alginate (*n* = 5; 8,1 %). Elf Teilnehmende (17,7 %) gaben an, antibiotikahaltige Salben zu verwenden.

Bei den Verbandswechseln wurden mehrheitlich Octenidin-haltige Desinfektionsmittel eingesetzt (*n* = 45; 72,6 %). Drei Teilnehmende (4,8 %) berichteten, regulär kein Desinfektionsmittel zu verwenden.

Der Wundverbandswechsel erfolgte überwiegend im Abstand von 2 Tagen (26/62; 41,9 %), gefolgt von einem Wechsel alle 3 Tage (19/62; 30,6 %), alle 4 bis 5 Tage (10/62; 16,1 %) sowie täglichen Wechseln (5/62; 8,1 %).

Vierzehn Teilnehmende führten elektive Débridements durch (14/62; 22,6 %). Der Einsatz von Kaltplasma wurde in 4 von 62 Fällen (6,5 %) angegeben, eine Vakuumtherapie (VAC) in 11 von 62 Fällen (17,7 %).

### Postoperative Wundinfektion

Das Vorgehen bei geschlossenen infizierten Wunden ist sehr heterogen. Zur Anwendung kommen Eröffnung der Wunde und/oder Spülung sowie der Einsatz einer systemischen Antibiotikatherapie. In 3 Fällen werden topische Antibiotikasalben verwendet (4,8 %). Bei dehiszenten offenen Wunden erhöht sich der Anteil auf 7 (11,3 %).

## Diskussion

Die vorliegende Umfrage zeigt eine ausgeprägte Heterogenität der postoperativen Wundversorgung in der Dermatochirurgie in Deutschland. Dies betrifft sowohl die Auswahl antiseptischer Substanzen als auch die eingesetzten Verbandsmaterialien und die Frequenz von Verbandswechseln. Die Ergebnisse spiegeln damit die bislang uneinheitliche Datenlage und das Fehlen klarer, speziell auf die Dermatochirurgie zugeschnittener Handlungsempfehlungen wider.

Internationale Leitlinien zur Prävention chirurgischer Wundinfektionen, veröffentlicht von der Weltgesundheitsorganisation (WHO), dem britischen National Institute for Health and Care Excellence (NICE) sowie den Centers for Disease Control and Prevention (CDC), geben unterschiedliche Empfehlungen zur präoperativen Hautdesinfektion [1–3]. Während WHO und NICE alkoholbasierte Lösungen in Kombination mit Chlorhexidin bevorzugen, empfiehlt das CDC allgemein alkoholbasierte Antiseptika. In der vorliegenden Umfrage wurden überwiegend alkoholbasierte sowie Octenidin-haltige Desinfektionsmittel eingesetzt, was mit den Ergebnissen aktueller Metaanalysen und Empfehlungen des Robert-Koch-Institutes übereinstimmt [4, 5]. Als „präoperativ“ wurde im Fragebogen die unmittelbar präinzisional im Operationssaal durchgeführte Hautantiseptik erfasst; nicht abgefragt wurden ggf. bereits Tage zuvor durchgeführte hygienische Vorbereitungsmaßnahmen.

In der Dermatochirurgie ist insbesondere bei länger andauernden Eingriffen der Einsatz eines Antiseptikums mit Remanenzeffekt aufgrund der anhaltenden antimikrobiellen Wirkung sinnvoll [5, 6]. Im schleimhautnahen Bereich erscheint der bevorzugte Einsatz mukosaverträglicher Antiseptika sinnvoll, da alkoholische Präparate ein erhöhtes Irritationspotenzial aufweisen. Chlorhexidin wird insbesondere im Bereich von Auge und Ohr aufgrund möglicher neuro- und ototoxischer Effekte kritisch bewertet und sollte bei freiliegendem Knorpel aufgrund potenzieller chondrotoxischer Effekte vermieden werden (s. auch Tab. 1).

Bemerkenswert ist, dass in der vorliegenden Umfrage von einzelnen Teilnehmenden gerade bei freiliegendem Knorpel oder exponierten Sehnen auf Polyhexanid-haltige Präparate ausgewichen wurde. Auch für Polyhexanid wird laut Herstellerangaben (z. B. B. Braun) von einer Anwendung auf freiliegendem Knorpelgewebe abgeraten, sodass dieses in der Praxis beobachtete Vorgehen kritisch zu hinterfragen ist [5, 7, 8].

Darüber hinaus konnte gezeigt werden, dass in einer In-vitro Studie auch Octenidin-haltige Desinfektionsmittel chondrotoxisch sind, nicht so Povidon-Jod [9].

Besondere Aufmerksamkeit verdient der Einsatz Octenidin-haltiger Lösungen in tiefen Wunden und Abszesshöhlen. Fallberichte beschreiben persistierende subkutane Ödeme und aseptische Fettgewebsnekrosen nach Anwendung in Wundhöhlen [10]. Octenidin darf daher nicht zur Spülung von Abszesshöhlen oder tiefen Wundtaschen eingesetzt werden und sollte aufgrund möglicher Interaktionen nicht mit anderen Antiseptika, wie z. B. Povidon-Jod, kombiniert werden.

Povidon-Jod scheint nur noch sehr selten standardmäßig eingesetzt zu werden. Aktuelle Daten sprechen dafür, dass die Mehrzahl der unter Povidon-Jod beobachteten Hautreaktionen irritativer Natur sind und nicht für eine echte allergische Sensibilisierung sprechen [11]. Dennoch erscheint diese Entwicklung vor dem Hintergrund an weiteren zur Verfügung stehenden Präparaten begründet. Außerdem ist zu bedenken, dass Povidon-Jod laut Fachinformation bei Patienten mit manifester oder latenter Hyperthyreose, autonomem Schilddrüsenadenom, Dermatitis herpetiformis Duhring sowie im zeitlichen Zusammenhang mit einer Radiojodtherapie nicht angewendet werden darf. Weiterhin ist bei Neugeborenen, Schwangeren, Stillenden, insbesondere bei großflächiger oder längerfristiger Anwendung aufgrund möglicher systemischer Jodresorption besondere Vorsicht geboten.

Zur praktischen Anwendung präoperativer Antiseptika s. auch Tab. 2.

**Tab. 2:** Praktische Anwendung präoperativer Antiseptika

Antiseptikum	Gegenanzeigen/Einschränkungen	Hinweise
Povidon-Jod-haltiges Desinfektionsmittel	Neugeborene, Säuglinge und Kleinkinder < 1 Jahr	Vergleichsweise lange Einwirkzeit (talgdrüsenarme Haut: 3 min, talgdrüsenreiche Haut: 10 min)
	Hyperthyreose oder andere manifeste Schilddrüsenerkrankungen	Potenziell wundheilungshemmend (zytotoxische Effekte auf Fibroblasten und Keratinozyten in vitro beschrieben)
	Dermatitis herpetiformis Duhring	Starke Verfärbung und Wirkungsabschwächung bei gleichzeitiger Anwendung mit Octenidin-haltigen Desinfektionsmitteln
	Vor, während und nach Radiojodtherapie	
	Gleichzeitige Anwendung mit quecksilberhaltigen Präparaten	
	Gleichzeitige Nutzung von Wasserstoffperoxid, Taurolidin oder silberhaltigen Desinfektionsmitteln oder Wundauflagen	
Octenidin-haltiges Desinfektionsmittel mit Phenoxyethanol	Verabreichungen unter Druck ins Gewebe	Einwirkzeit: ca. 1 min (produktspezifische Fachinformation beachten)
	Perforiertes Trommelfell oder freiliegender Knorpel	Geeignet für schleimhautnahe Areale
	Frühgeborene mit niedrigem Geburtsgewicht berichtet	Starke Verfärbung und Wirkungsabschwächung bei gleichzeitiger Anwendung mit Povidon-Jod
		Nicht zur Spülung tiefer Wundhöhlen oder Abszesshöhlen geeignet
Octenidin-haltiges Desinfektionsmittel mit Propranolol	Nähe zu Augen oder Schleimhäuten	Einwirkzeit: ca. 15–60 s, vollständige Abtrocknung abwarten (produktspezifische Fachinformation beachten)
	Offene Wunden	Aufgrund des hohen Alkoholanteils nicht bei Frühgeborenen und Neugeborenen mit unreifer Haut anwenden
	Perforiertes Trommelfell oder freiliegender Knorpel	Vollständige Abtrocknung vor Einsatz elektrochirurgischer Instrumente aufgrund Brandgefahr
Chlorhexidin-haltiges alkoholisches Desinfektionsmittel	Nähe zu Augen oder Schleimhäuten	Einwirkzeit: ca. 30 s bis 3 min je nach Hersteller, bis zur vollständigen Abtrocknung (produktspezifische Fachinformation beachten)
	Offene Wunden	Vollständige Abtrocknung vor Einsatz elektrochirurgischer Instrumente aufgrund Brandgefahr
	Perforiertes Trommelfell	
	Direkter Kontakt mit neuralem Gewebe	

In der vorliegenden Umfrage wurden hypochlorige Lösungen von keinem der Teilnehmenden als Standardantiseptikum angegeben. Auch silberhaltige Wundprodukte wurden im Fragebogen nicht separat erfasst; sie werden in aktuellen Übersichtsarbeiten insbesondere für kritisch kolonisierte oder chronische Wunden diskutiert, wobei die Evidenz für einen generellen Vorteil bei akuten postoperativen Wunden begrenzt bleibt. Beide Stoffgruppen sollten in künftigen Erhebungen gezielt berücksichtigt werden.

Studien und Metaanalysen, die den zeitlichen Abstand bei Verbandswechseln bis zum Fadenzug untersucht haben, konnten keinen klaren Unterschied finden [12–14]. Das mindestens 48-stündige Belassen des initialen Verbands, falls nicht kontaminiert, erscheint sinnvoll, um die initiale Wundheilung nicht negativ zu beeinflussen. Die Anzahl an folgenden Wechseln sollte so gering wie möglich gehalten werden. Ein deutlich längeres Belassen eines konventionellen Wundverbandes > 48 h ist jedoch wieder mit einem Anstieg des Infektionsrisikos assoziiert [15]. Moderne transparente semipermeable Folienverbände mit Polyurethanschaum scheinen längere Verbandswechselintervalle zu erlauben, sofern keine relevante Wundsekretion vorliegt [16].

Für zweizeitige chirurgische Vorgehen existieren bislang keine spezifischen Leitlinienempfehlungen zur Bewertung unterschiedlicher Wundauflagen; entsprechende Evidenz liegt in der Literatur nicht vor. Vor diesem Hintergrund erscheint der routinemäßige Einsatz kostenintensiver Wundauflagen derzeit nicht gerechtfertigt. Da die Fragebogenformulierung nicht zwischen temporärer Defektdeckung im Rahmen zweizeitiger Eingriffe und permanentem Hautersatz differenzierte, ist die vereinzelte Nennung von Integra® als mögliche Fehlzuordnung im Sinne einer Unschärfe der Fragestellung zu werten.

Die weiterhin relevante Anwendung topischer Antibiotika in der postoperativen Wundversorgung ist vor dem Hintergrund zunehmender Resistenzentwicklungen kritisch zu bewerten. Insbesondere die Zunahme von gegen Fusidinsäure und Mupirocin resistenten *Staphylococcus-aureus*-Klonen ist gut dokumentiert [17, 18]. Auch aktuelle Analysen aus großen Gesundheitssystemen zeigen eine relevante Prävalenz von Resistenzgenen gegenüber topischen Antibiotika und Antiseptika [19]. Wunden mit sekundärer Wundheilung nach dermatochirurgischen Eingriffen sind verglichen mit einem primären Wundverschluss gemäß den Ergebnissen systematischer Übersichtsarbeit nicht mit einem höheren Risiko für Wundinfektionen assoziiert [20]. Die routinemäßige Anwendung topischer Antibiotika bei primär nicht infizierten Wunden ist daher nicht evidenzbasiert und sollte vermieden werden.

Im vorliegenden Fragebogen wurde nicht erfasst, welche topischen Antibiotika (z. B. Fusidinsäure- oder Mupirocin-haltige Präparate) konkret verwendet wurden. Bei klinisch manifest infizierten Wunden, definiert durch lokale Infektionszeichen wie Rötung, Überwärmung, Schmerz, Sekretion oder Fieber, kann eine gezielte antimikrobielle Therapie – bevorzugt systemisch nach Erregernachweis – indiziert sein; eine routinemäßige topische Antibiotikagabe ist aufgrund der beschriebenen Resistenzproblematik nicht generell zu empfehlen. Eine standardisierte, allgemein anerkannte Definition der „lokal infizierten Wunde“ existiert für die Dermatochirurgie bislang nicht, was die Vergleichbarkeit der Umfrageergebnisse zusätzlich einschränkt.

Eine differenzierte, im Fragebogen vordefinierte Klassifikation (z. B. nach lokaler vs. systemischer Infektion) lag nicht vor; die Einordnung als „infizierte Wunde“ oblag der subjektiven Einschätzung der Teilnehmenden.

Bei der Versorgung sekundär heilender Wunden zeigte sich eine besonders hohe Variabilität der eingesetzten Verbandsmaterialien. Die Evidenz zur Überlegenheit einzelner Verbandstypen ist limitiert. Cochrane-Analysen konnten keinen eindeutigen Vorteil bestimmter Wundauflagen zur Prävention postoperativer Infektionen nachweisen [21]. Netzwerkmetaanalysen deuten zwar auf potenzielle Vorteile einzelner moderner Verbandsmaterialien hin, die Aussagekraft dieser Daten ist jedoch aufgrund methodischer Limitationen eingeschränkt [22]. Schaumverbände scheinen im Vergleich zu Gazen Vorteile hinsichtlich Schmerzlinderung und Patientenzufriedenheit zu bieten [23].

Individuell intraoperativ angefertigte, nichtadhäsive Wundauflagen bei sekundär heilenden Wunden nach radikaler Exzision scheinen eine effektive, schmerzarme und zugleich deutlich kostengünstigere Alternative zu kommerziell verfügbaren Wundauflagen darzustellen [24].

Angaben zu Anwendungsdauer, Therapieziel und klinischem Ergebnis einer Vakuumtherapie wurden im Fragebogen nicht erhoben und können daher nicht berichtet werden.

Der routinemäßige Einsatz von Tamponaden bei Abszessen kleiner als 5 cm zeigte in Studien keinen Vorteil hinsichtlich Wundheilung oder Reinterventionsrate, führte jedoch zu einer erhöhten Schmerzbelastung [25]. Eine differenzierte Indikationsstellung erscheint daher sinnvoll (Tab. 3).

**Tab. 3:** Übersicht evidenzbasierter Empfehlungen zur prä- und postoperativen Wundversorgung in der Dermatochirurgie mit zugeordnetem Evidenzgrad

Bereich	Empfehlung	Evidenzgrad [Quelle]
Präoperative Hautantiseptik	Alkoholbasiertes Antiseptikum, ggf. in Kombination mit Chlorhexidin oder Octenidin; Remanenzeffekt bei längeren Eingriffen vorteilhaft	III [1–3]
Schleimhautnahe/mukosanahe Areale	Mukosaverträgliche, nichtalkoholische Antiseptika (z. B. Octenidin); Meidung stark irritierender Alkoholpräparate	II [5]
Freiliegender Knorpel/exponierte Sehnen	Chlorhexidin und ggf. auch Polyhexanid sowie Octenidin vermeiden (chondrotoxisches Potenzial); Povidon-Jod als Alternative erwägen	I/II [7–9]
Abszesshöhlen/tiefe Wundtaschen	Octenidin nicht zur Spülung verwenden (Risiko für Fettgewebsnekrosen); Alternativpräparate, z. B. Povidon-Jod, bevorzugen	III [10]
Primärer Wundverschluss – Verbandsdauer	Verband für mindestens 48 h belassen, sofern keine Sekretion/Infektion/Kontamination vorliegt; Wechselfrequenz so gering wie möglich halten	I [12–15]
Sekundär heilende Wunden – Verbandsmaterial	Kein eindeutiger Wirksamkeitsvorteil einzelner Wundauflagen belegt; Schaumverbände ggf. vorteilhaft bezüglich Schmerz/Zufriedenheit	I [21–23]
Topische Antibiotika	Kein routinemäßiger Einsatz bei primär nicht infizierten Wunden (Resistenzentwicklung, insbesondere Fusidinsäure/Mupirocin)	II [17–20]
Tamponade kleiner Abszesse (< 5 cm)	Kein genereller Vorteil für Wundheilung/Reinterventionsrate; erhöhte Schmerzbelastung – differenzierte Indikationsstellung	I [25]

I = geringe Evidenz, II = mittlere Evidenz, III = hohe Evidenz

Der in der vorliegenden Umfrage berichtete Einsatz von Kaltplasma bei sekundär heilenden Wunden (4/62; 6,5 %) spiegelt eine noch zurückhaltende Verbreitung dieser Technologie in der Dermatochirurgie wider. Erste klinische Studien deuten auf eine antimikrobielle und wundheilungsfördernde Wirkung von Kaltplasma hin, belastbare Daten speziell für postoperative dermatochirurgische Wunden fehlen jedoch bislang, sodass der Stellenwert dieses Verfahrens aktuell nicht abschließend beurteilt werden kann. Perspektivisch erscheint die Etablierung prospektiver, multizentrischer Studien mit standardisierten Outcome-Parametern erforderlich, um evidenzbasierte, Dermatochirurgie-spezifische Empfehlungen sowohl für die prä- als auch die postoperative Wundversorgung zu entwickeln. Die vorliegenden Ergebnisse können hierfür als Grundlage für die Entwicklung und Priorisierung entsprechender Konsensusempfehlungen der Deutschen Gesellschaft für Dermatochirurgie dienen.

Limitationen der Studie ergeben sich aus dem explorativen Studiendesign sowie dem Einsatz eines nicht validierten Fragebogens, da bislang kein etabliertes Instrument zur Erfassung der postoperativen Wundversorgung in der Dermatochirurgie zur Verfügung steht. Zudem ist ein Selektionsbias nicht auszuschließen, da insbesondere besonders motivierte oder an dem Thema interessierte operativ tätige DermatologInnen an der Umfrage teilgenommen haben könnten. Die insgesamt geringe Anzahl vollständig ausgefüllter Fragebögen schränkt die Generalisierbarkeit der Ergebnisse zusätzlich ein und unterbindet eine Subgruppenanalyse. Darüber hinaus wurden keine klinischen Outcome-Parameter erhoben, sodass ein direkter Zusammenhang zwischen den berichteten Versorgungsstrategien und klinischen Ergebnissen nicht bewertet werden kann. Mit einer Rücklaufquote von 4,5 % (62/1367 versendete Fragebögen) liegt der Anteil vollständig beantworteter Fragebögen deutlich unter dem für Online-Umfragen dieser Art üblichen Bereich; dies schränkt die Repräsentativität und Generalisierbarkeit der Ergebnisse zusätzlich ein. Einzelne Fragen des insgesamt 26 Items umfassenden Fragebogens (z. B. zur Art der eingesetzten Wundauflage bei Abszesshöhlen oder zur alloplastischen Defektdeckung) waren zudem nicht hinreichend trennscharf formuliert, sodass die entsprechenden Ergebnisse nur eingeschränkt interpretierbar sind; ein Teil der zugrunde liegenden Einzelantworten (u. a. zu Fragen des primären Wundverschlusses) konnte aufgrund der hohen Heterogenität der Angaben zudem nicht in strukturierter Form dargestellt werden. Der inhaltliche Schwerpunkt der Arbeit liegt auf antimikrobiellen und verbandstechnischen Aspekten der postoperativen Wundversorgung; weitere relevante Aspekte der dermatochirurgischen Wundbehandlung, etwa die Versorgung von Spalthautentnahmestellen, wurden im Rahmen dieser Erhebung nicht gesondert erfasst und sollten Gegenstand künftiger Untersuchungen sein. Eine Folgeerhebung mit einem gezielt vereinfachten, stärker standardisierten Fragebogen erscheint vor diesem Hintergrund sinnvoll, um belastbarere und besser vergleichbare Daten zu generieren. Dennoch liefert die Umfrage einen realistischen Einblick in die aktuelle Versorgungspraxis und unterstreicht den Bedarf an standardisierten, evidenzbasierten Empfehlungen für die Dermatochirurgie.

## Fazit für die Praxis

- Der Einsatz topischer Antibiotika in der Dermatochirurgie sollte aufgrund zunehmender Resistenzentwicklungen restriktiv erfolgen, insbesondere bei primär nicht infizierten Wunden.
- Alkoholbasierte und Octenidin-haltige Antiseptika werden in der Praxis häufig eingesetzt; deren Anwendung sollte indikationsgerecht und unter Berücksichtigung möglicher Nebenwirkungen erfolgen.
- Octenidin-haltige Lösungen sollten aufgrund des Risikos für Fettgewebsnekrosen nicht zur Spülung von Abszesshöhlen oder tiefen Wundhöhlen verwendet werden.
- Der primäre Wundverband kann für mindestens 48 h belassen werden, sofern keine Anzeichen von Sekretion, Infektion oder Kontamination vorliegen.
- Bei Patientinnen und Patienten ohne Diabetes mellitus und ohne Immunsuppression ist eine routinemäßige Tamponade kleiner Abszesse kritisch zu hinterfragen.
- Die Entwicklung klarer, für die Dermatologie spezifischer Handlungsempfehlungen zur postoperativen Wundversorgung erscheint dringend erforderlich.

## Data Availability

Die in dieser Studie erhobenen Datensätze können auf begründete Anfrage beim Korrespondenzautor angefordert werden.
